# Assessment of methylcitrate and methylcitrate to citrate ratio in dried blood spots as biomarkers for inborn errors of propionate metabolism

**DOI:** 10.1038/s41598-019-48885-9

**Published:** 2019-08-26

**Authors:** Osama Y. Al-Dirbashi, Majid Alfadhel, Khalid Al-Thihli, Nahid Al Dhahouri, Claus-Dieter Langhans, Zalikha Al Hammadi, Aisha Al-Shamsi, Jozef Hertecant, Jürgen G. Okun, Georg F. Hoffmann, Fatma Al-Jasmi

**Affiliations:** 1 College of Medicine and Health Sciences, UAE University, Al-Ain, UAE; 2 Newborn Screening Ontario, Children’s Hospital of Eastern Ontario, Ottawa, ON Canada; 3 Department of Pediatrics, University of Ottawa, Ottawa, ON Canada; 4 Research Institute, Children’s Hospital of Eastern Ontario, Ottawa, ON Canada; 5 Division of Genetics, Department of Pediatrics, King Abdullah Specialized Children’s Hospital, Ministry of National Guard Health Affairs, Riyadh, Saudi Arabia; 6 King Abdullah International Medical Research Centre, King Abdulaziz Medical City, Ministry of National Guard Health Affairs, Riyadh, Saudi Arabia; 7 King Saud bin Abdulaziz University for Health Sciences, Ministry of National Guard Health Affairs, Riyadh, Saudi Arabia; 8 Genetic and Developmental Medicine Clinic, Sultan Qaboos University Hospital, Muscat, Sultanate of Oman; 9 Department of General Pediatrics, Division of Neuropediatrics and Metabolic Medicine, Center for Pediatric and Adolescent Medicine, University Hospital Heidelberg, Heidelberg, Germany; 10 Department of Genetics, Latifa Hospital, Dubai, United Arab Emirates; 11 Department of Pediatrics, Tawam Hospital, Al-Ain, United Arab Emirates

**Keywords:** Biochemistry, Predictive markers

## Abstract

Deficiency of propionyl-CoA carboxylase causes propionic acidemia and deficiencies of methylmalonyl-CoA mutase or its cofactor adenosylcobalamin cause methylmalonic acidemia. These inherited disorders lead to pathological accumulation of propionyl-CoA which is converted in Krebs cycle to methylcitrate (MCA) in a reaction catalyzed by citrate synthase. In healthy individuals where no propionyl-CoA accumulation occurs, this enzyme drives the condensation of acetyl-CoA with oxaloacetate to produce citric acid (CA), a normal Krebs cycle intermediate. The competitive synthesis of CA and MCA through the same enzymatic mechanism implies that increase in MCA production is accompanied by decrease in CA levels. In this study, we assessed MCA concentration and the ratio of MCA/CA as plausible markers for propionic and methylmalonic acidemias. We measured MCA and CA in dried blood spots using liquid chromatography tandem mass spectrometry. The reference ranges of MCA, CA and MCA/CA in 123 healthy individuals were ≤0.63 µmol/L, 36.6–126.4 µmol/L and 0.0019–0.0074, respectively. In patients with propionic and methylmalnic acidemias (n = 7), MCA concentration ranged between 1.0–12.0 µmol/L whereas MCA/CA was between 0.012–0.279. This is the first report to describe the potential role of MCA and MCA/CA in dried blood spots as diagnostic and monitoring biomarkers for inherited disorders of propionyl-CoA metabolism.

## Introduction

Propionic acidemia (PA) and methylmalonic acidemia (MMA) are inborn errors of propionyl-CoA metabolism caused by defects in enzymes required for correct catabolism of branched-chain amino acids, odd chain fatty acids and cholesterol^1^. Mutations in genes encoding propionyl-CoA carboxylase, a biotin-requiring enzyme that catalyzes the conversion of propionyl-CoA to methylmalonyl-CoA cause PA^2,3^. MMA is a more etiologically heterogeneous disorder that can present as isolated MMA or in combination with homocystinuria. The former is caused by genetic defects in methylmalonyl-CoA mutase or its cofactor adenosylcobalamin. Combined MMA and homocystinuria is due to genetic anomalies affecting adenosylcobalamin and methylcobalamin, the two biologically active forms of vitamin B12. Other less common causes of MMA have also been reported^1,4,5^.

Patients with PA and MMA typically present in the neonatal period with high anion gap metabolic acidosis, ketosis, and hyperammonemia. Severe life-threatening complications including progressive neurological dysfunction or early death are common^6–8^. Early diagnosis is critical as presymptomatic treatment is associated with better outcomes^9,10^. The biochemical hallmarks of PA include accumulation of glycine, propionylcarnitine, propionylglycine, 3-hydroxypropionic acid, and methylcitrate (MCA). In MMA, accumulation of methylmalonic acid together with elevated glycine, propionylcarnitine, 3-hydroxypropionic acid and MCA is diagnostic^1,3,11^.

Management of PA and MMA aims at reducing toxic metabolites and maintaining normal growth and development. Protein restriction, optimal calorie intake, carnitine supplementation and suppressing microbial propionate production represent the treatment mainstay. Some MMA patients are vitamin B_12_ responsive and benefit from hydroxycobalamin treatment. Monitoring methylmalonic acid levels in these patients is an essential part of the management plan. Regrettably, a suitable biochemical marker to monitor PA patients is currently lacking.

Recently we developed methods for the analysis of MCA in dried blood spot (DBS) and dried urine spot samples and showed that this marker improves newborn screening for disorders of propionate metabolism^12–14^. MCA and its physiological counterpart, citric acid (CA) are produced in Krebs cycle in a reaction catalyzed by the same enzyme, namely citrate synthase^15,16^. In this work, we used liquid chromatography tandem mass spectrometry (LC-MS/MS) to simultaneously determine CA and MCA in DBS in the same analytical run. This allowed for the calculation of MCA/CA ratio, a novel biomarker that may potentially apply for all disorders of propionate metabolism. The diagnostic and monitoring potential of these biomarkers was evaluated using specimens collected from controls and patients with established diagnosis of PA and MMA.

## Results

### Sample analysis

**Figure 1:**
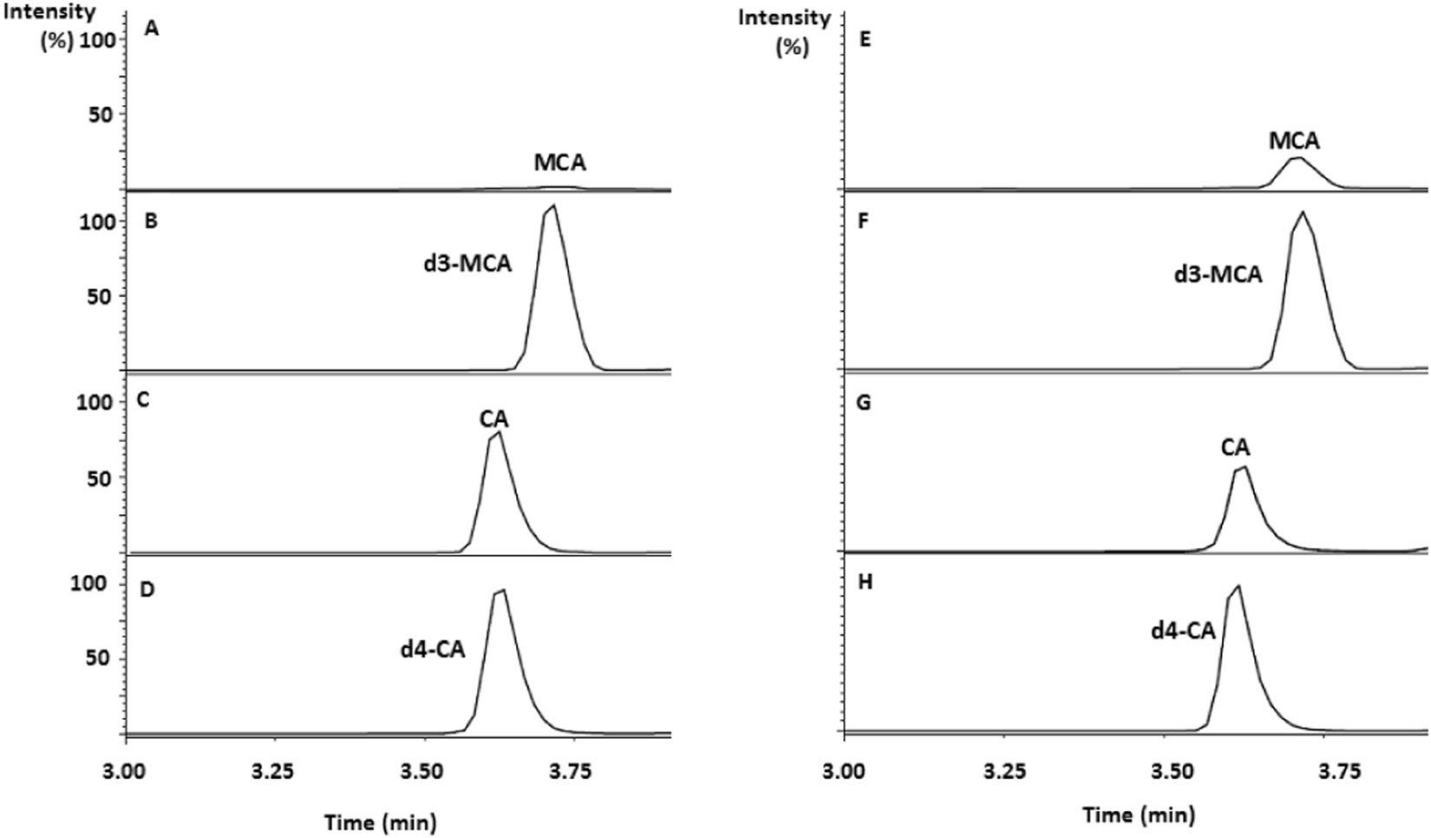
Extracted mass chromatograms obtained with a DBS from healthy individual (**A**–**D**), and from a patient with MMA (**E**–**H**).

Optimum derivatization conditions for CA and its deuterium labeled internal standard (d4-CA) with 4-[2-(N,N-dimethylamino)ethylaminosulfonyl]-7-(2-aminoethylamino) -2,1,3-benzoxadiazole (DAABD-AE) were similar to those previously reported^12,14,17,18^. Infusion of the derivatization reaction mixture into the first quadrupole revealed intense ions at mass to charge ratio (*m/z*) of 485 and 489 corresponding to DAABD-AE derivatives of CA and d4-CA. Collision induced dissociation and subsequent scanning by the second resolving quadrupole revealed *m/z* 151 as an intense and common fragment to both CA and d4-CA. These results are in agreement with the fragmentation pattern obtained with MCA and its deuterium labeled analogue d3-MCA^12^. Chromatographic conditions were optimized so that CA and MCA derivatives are eluted away from other substances with potential ion suppression effect. CA and MCA eluted at 3.6 and 3.7 min, respectively. Surging the organic content to 95% was required to wash out late eluting compounds from the column. After each sample, the column was re-equilibrated for 2 min giving an injection-to-injection time of 8 min. Figure 1 shows extracted mass chromatograms obtained with a DBS sample from a healthy individual (Fig. 1A–D), and a DBS sample from a patient with MMA (Fig. 1E–H).

### Assay performance

Linear relationships between the area ratios of analyte to internal standard *versus* concentration in DBS were established by spiking blood in the range of 2.5–200 µmol/L for CA and 0.25–16 µmol/L for MCA. Given that endogenous CA was at 92.8 µmol/L, the overall linearity range (endogenous + spiked) was up to 292.8 µmol/L. For MCA, the endogenous concentration was 0.23 µmol/L, therefore and the overall linearity range (endogenous + spiked) was up to 16.23 µ µmol/L. Linear regression equations for CA and MCA of *y* = 0.0065*x*-0.0041 (*r* = 0.995) and *y* = 0.0046*x* + 0.0009 (*r* = 0.997), respectively, were obtained with *y* as peak area ratio, *x* as the added analyte concentration (µmol/L) and *r* as the correlation coefficient.

Accuracy, intraday (n = 12) and interday (n = 12) imprecisions of CA and MCA in spiked DBS specimens are shown in Table 1. The analytical recovery calculated from these measurements ranged between 98–105% for MCA and 95–110% for CA.

**Table 1:** Accuracy, within-day and between-day imprecision of CA and MCA in DBS spiked at two different levels.

Sample	Analyte	Concentration added, µmol/L	Within-day (n = 12)				Between-day (n = 12)			
			Mean, µmol/L	SD^a^, µmol/L	CV^b^, %	Accuracy, %	Mean, µmol/L	SD, µmol/L	CV, %	Accuracy, %
QC 1^c^	CA	115	114	6.7	5.9	−0.9	126	11.1	8.8	9.6
	MCA	1.9	2.0	0.19	9.9	5.3	2.0	0.23	11.8	5.3
QC 2	CA	200	191	15.4	8.1	−4.5	199	24.7	12.4	−0.5
	MCA	5.7	5.6	0.53	9.4	−1.8	5.9	0.53	9.1	3.5

^a^SD = standard deviation.

^b^CV (%) = coefficient of variation.

^c^QC = quality control.

### Analysis of controls and patients’ samples

**Figure 2:**
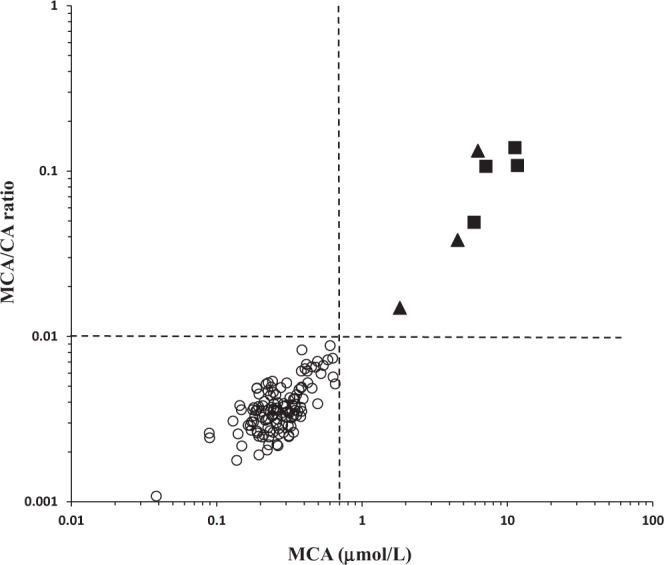
Distribution of MCA and the MCA/CA ratio in DBS from the study population. Open circles represent controls, solid triangles represent MMA and solid squares represent PA. The dashed lines represent arbitrary cutoffs of MCA and MCA/CA ratio of 0.7 μmol/L and 0.01, respectively.

**Figure 3:**
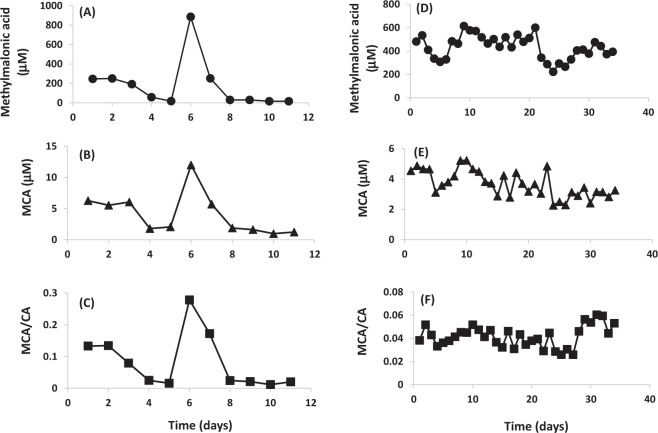
Methylmalonic acid (**A**), MCA (**B**) and MCA/CA (**C**) in daily consecutive DBS samples (n = 11) collected from a vitamin B12 responsive MMA patient before and after starting treatment with IM hydroxycobalamin (1.0 mg/day). Methylmalonic acid (**D**), MCA (**E**) and MCA/CA (**F**) in daily consecutive DBS samples (n = 34) collected from a vitamin B12 non-responsive MMA patient before and after starting treatment with IM hydroxycobalamin (1.0 mg/day).

Table 2 shows the median, range and 95% reference ranges of CA, MCA and MCA/CA ratio in DBS from controls (n = 123) and patients (n = 7) with confirmed inborn error of propionate metabolism. Figure 2 shows the distribution of MCA and the ratio of MCA/CA in the study population. Figure 3 shows the results of MCA and MCA/CA measured in consecutive DBS samples collected daily from two MMA patients before and after starting IM hydroxycobalamin (1.0 mg/day). Shown for comparison purpose, methylmalonic acid was also analyzed according to known methods^19^.

**Table 2:** MCA, CA and MCA/CA in DBS from patients and controls as measured by the current method.

Sample	Parameter	MCA (μmol/L)	CA (μmol/L)	MCA/CA
Patients (n = 50)^a^	Median	3.7	53.2	0.043
	Range	1.0–12.0	26.2–89.1	0.012–0.279
Controls (n = 123)	Median	0.26	73.6	0.0036
	Reference interval^b^	ND^c^- 0.63	36.6–126.4	0.0019–0.0074

^a^These 50 DBS were collected from 7 patients on different days and represent various clinical setting.

^b^2.5–97.5% reference interval.

^c^ND: not detectable.

## Discussion

Propionylcarnitine, a diagnostic biomarker common to both PA and MMA, is not recommended as follow up marker to guide clinical patient management^6^. While ammonia, blood gases and calculated anion gap are non-specific markers that also occur in other disorders^1,3,6^, a number of complications occur in PA and MMA patients despite these biomarkers being within therapeutic targets. Measuring methylmalonic acid, unique to MMA, provides important clues on response to treatment, vitamin B_12_ responsiveness and compliance^20^. On the other hand, the challenge in managing PA patients is confounded by the lack of a reliable biomarker for monitoring and prognostication.

Formed as a consequence of propionyl CoA accumulation, MCA is a characteristic abnormal metabolite that is common to all inborn errors of propionyl-CoA metabolism including PA and MMA. Citrate synthase, the enzyme catalyzing the physiological production of CA in Krebs cycle is responsible of forming MCA as a result of condensation of the pathologically accumulating propionyl CoA with oxaloacetate^15,16^. Elevated MCA and deficient CA are implicated in the pathogenesis of MMA and PA. Brain cell cultures derived from wild type mouse embryos challenged with MCA showed alterations of the developing brain morphology, increased ammonium concentrations and apoptotic cell death^21,22^. Given the role of propionate metabolism in anaplerosis of the citric acid cycle through provision of succinyl-CoA and sequestration of oxaloacetate by propionyl-CoA, severe deficiency of citric acid cycle intermediates is anticipated in patients with defects of propionate metabolism^3^. A biomarker that takes these observations into account, should arguably be more clinically useful.

In a previous work, we reported a novel method for the analysis of MCA in neonatal DBS specimens using LC-MS/MS, a technology commonly available in clinical laboratories^12,13^. In the current work, we extended our method to include the simultaneous analysis of CA, the structural and biological counterpart of MCA with implications for the management of patients with PA and MMA^23^. Given the physicochemical similarities of these tricarboxylic acids, CA was also derivatized with DAABD- AE using the conditions established earlier for MCA^12,14^. The sample preparation was minimal and consisted of a single step encompassing both extraction and derivatization. The resultant DAABD derivatives of CA and MCA were well resolved by reversed phase chromatography in less than 4 min (Fig. 1). The reference range of CA in DBS obtained in this work of 36.6–126.4 µmol/L was comparable to that previously reported in plasma of 128.2 ± 31.1µmol/L^24^. In DBS samples from patients with inborn error of propionate metabolism, CA that ranged from 26.2–89.1 µmol/L was significantly lower than controls (*p* < 0.01). Our assay was adequately reproducible with CV (%) measured at various concentrations of ≤15.1% (Table 1). The use of deuterium labelled analogues as internal standards improved the overall performance of this assay.

The study presented here is the first to evaluate the potential of MCA and its ratio to CA as monitoring markers of disorders of propionate metabolism. The competitive synthesis of CA and MCA through the same enzymatic mechanism implies that the increase in MCA production is accompanied by a decrease in CA levels. Therefore, this inverse relation disrupts the resultant MCA/CA ratio and may serve as an important indictor to assess the disease course and/or severity. As a proof of concept, we assessed MCA and MCA/CA as monitoring biomarkers in consecutive DBS samples from two MMA patients, one known to be vitamin B_12_ responsive and the other is vitamin B_12_ non-responsive. We compared our results with methylmalonic acid, the routinely used follow up marker (Fig. 3). The vitamin B12 responsive patient was initially admitted due to metabolic decompensation caused by non-compliance with the prescribed therapy. As part of the intervention, the patient received daily IM hydroxycobalamin (1.0 mg). DBS samples were collected at admission and daily thereafter for a total of 11 days. As shown in Fig. 3A–C, MCA and MCA/CA ratio, adequately predicted the response to treatment in the first five days as well as the metabolic derangement and consequent accumulation of MCA which was triggered by a nosocomial infection (day 6 and 7). This pattern was in agreement with that obtained with methylmalonic acid. In the patient who is non-responsive to vitamin B12 treatment (Fig. 3D–F), methylmalonic acid, MCA and MCA/CA ratio remained elevated despite the daily administration of hydroxycobalamin (1.0 mg IM). One may argue that MCA and MCA/CA are potentially superior to MMA as routine biomarkers due to the simple sample preparation required and universal applicability to all inborn errors of propionate metabolism including PA.

Although MCA alone seems adequate to discriminate patients from controls as shown in Fig. 2, the MCA/CA ratio proposed in this work might be superior as it can be conveniently calculated using instrument readout without the need for demanding calibration curve procedures. Further, measuring both analytes in the same run improves the analytical process and compensates for instrumental or sample preparation variations. We also believe that the use of MCA/CA ratio that depends on two competitively-synthesized and inversely-related markers may allow for improved ascertainment of patients with milder disease variants associated with subtle MCA elevations.

Nevertheless, to establish these markers in DBS as predictors of disease course and/or therapeutic outcomes, additional studies are required to assess their clinical validity and utility using larger sample size from patients with other relevant disorders, and across different pathophysiological circumstances.

In conclusion, this is the first report on the use of MCA and its ratio to CA as novel markers with potential diagnostic and monitoring roles in patients of inherited disorders of propionyl-CoA metabolism. Stratification of MCA levels using CA as denominator provides valuable clues on PA and MMA as these inversely related metabolites are competitively synthesized by the same enzyme system. The combined use of MCA concentration and the ratio of MCA stratified with CA significantly improved the specificity of our method while maintaining 100% sensitivity. The performance of MCA and its ratio to CA as follow up markers for our MMA patients was comparable to that of the routinely used methylmalonic acid. These novel biomarkers could prove more versatile as they can be used also in PA patients. After validation with more patients’ samples, our simple and robust approach can be efficiently included as part of care of patients with inherited propionyl-CoA metabolic defects.

## Methods

### Chemicals

Cambridge Isotopes Laboratories (Tewksbury, Massachusetts, USA) was the source of MCA, d3-MCA and d4-CA. Merck (Darmstadt, Germany) supplied us with water, acetonitrile and methanol (LC-MS/MS grade). The following chemicals were purchased from Sigma Aldrich (Taufkirchen, Germany): CA, perfluorooctanoic acid (PFOA), 4-(dimethylamino) pyridine (DMAP), DAABD-AE and N-(3-dimethylaminopropyl)-N′-ethylcarbodiimide hydrochloride (EDC). Stock solutions of CA, MCA and the deuterium labeled internal standards were prepared in acetonitrile:water (1:1, *v/v*) at 1.0 mg/mL and diluted to the desired concentrations using the same solvent. These solutions were protected from light and stored at −20 °C for 6 months as previously described^14^.

### Study samples

Al Ain Medical District Human Research Ethics Committee granted approval for this study (ERH-2017–5494 17-01). All experiments were carried out according to applicable rules and regulations. Participants, parents or legal guardians provided informed consent for study participation. For the determination of reference ranges, DBS samples from control subjects (n = 123) were analyzed. DBS samples (n = 50) from seven confirmed patients with MMA (n = 3) and PA (n = 4) were also studied. Of these fifty, consecutive daily DBS specimens before and after starting hydroxycobalamin treatment (1.0 mg/day intramuscular injection, IM) were collected from MMA patient-1 (n = 11) and MMA patient-2 (n = 34). DBS specimens were prepared by spotting 70 µL of blood on Whatman 903 filter paper cards using an Eppendorf pipette. These specimens were dried at ambient temperature for at least 4 hours and placed in sealed plastic bags with desiccant at 4 °C until analysis.

### Sample pretreatment

CA and MCA were measured in DBS samples as derivatives of DAABD-AE using a modified version of our previously described method^12^. In brief, a 3.2 mm circle was excised from the DBS specimen, placed in an Eppendorf Snap-Cap microcentrifuge tube (1.5 mL) and incubated for 45 min at 65 °C with the following: 20 µL of d3-MCA and d4-CA (24 µmol/L in 50% acetonitrile) and 100 µL of a mixture of 25 mmol/L EDC in water, 2 mmol/L DAABD-AE in 90% acetonitrile, and 25 mmol/L DMAP in acetonitrile (1:2:1,*v/v/v*) as we previously described^14^. After adding 900 μL of methanol:water (1:9, *v/v*) containing 0.5 g/L of PFOA to stop the reaction, 2 µL of the mixture were injected into the LC-MS/MS system.

### LC-MS/MS analysis

In this work, we used our previously described^14^ LC-MS/MS system which consists of a Nexera X2 UHPLC interfaced with an LC-MS 8060 triple quadrupole mass spectrometer equipped with an electrospray ion source (Shimadzu, Kyoto, Japan). Chromatography was accomplished using an Acquity UPLC BEH C18 column (2.1 × 50 mm, 1.7 µm, Waters, Milford, USA) maintained at 40 °C during analysis.

Chromatographic analysis of CA, MCA and their stable isotope labeled internal standards was achieved using 2% methanol containing 0.5 g/L PFOA for 4 min as mobile phase. The methanol concentration was increased to 85% at 4 min and to 95% at 6 min using linear gradient. A 2 min column re-conditioning step with 2% methanol containing 0.5 g/L PFOA was included after each injection. A constant flow rate of 400 μL/min was used in all experiments.

Analysis by MS/MS was in the positive ion mode using argon as collision gas with collision energy of 22 eV, capillary voltage of 3.4 kV and cone voltage of 35 V. Desolvation temperature of 120 °C and ion source temperature of 350 °C were applied. Detection was in the multiple reaction monitoring mode with *m/z* of 485 > 151 and 489 > 151 for CA and d4-CA, respectively. MCA and d3-MCA were detected using *m/z* 499 > 151 and 502 > 151 as previously described^12,14^.

### Method validation

DBS calibrators used to create the standard curves were prepared using control blood spiked with CA and MCA in the range of 2.5–200 µmol/L and 0.25–16 µmol/L, respectively. Unspiked DBS samples were included in the analysis to account for analyte concentrations that present naturally in blood. To determine the intraday (n = 12) and interday (n = 12) variations, quality control DBS samples spiked with target analytes (Table 1) were used. To assess the analytical recovery, we analyzed DBS samples before and after enrichment at known analyte concentrations. Accuracy expressed as % error was determined from DBS specimens containing known analyte concentrations using the following formula: [Accuracy (%) = 100 × (concentration measured – concentration added)/concentration added].

## Data Availability

The data that support the findings of this study are available within the manuscript. If required, additional data will be made available on reasonable request from the corresponding author [O.Y.A.].
